# Impact of Physician Specialty on Treatment Costs of Invasive Melanoma

**DOI:** 10.3390/diseases12110284

**Published:** 2024-11-08

**Authors:** John P. Garcia, Olivia A. Ho, Syed Ali Haider, Sahar Borna, Cesar A. Gomez-Cabello, Antonio Jorge Forte, Aaron C. Spaulding

**Affiliations:** 1 Division of Plastic Surgery, Mayo Clinic, Jacksonville, FL 32224, USA; 2 Center for Digital Health, Mayo Clinic, Rochester, MN 55905, USA; 3 Department of Health Science Research, Mayo Clinic, Jacksonville, FL 32224, USA

**Keywords:** Melanoma, skin cancer, healthcare cost, physician specialty, dermatology, general surgery, plastic surgery

## Abstract

Introduction: Melanoma is a deadly type of skin cancer that develops from melanocytes and can manifest on the skin or other regions of the body. Its incidence is increasing rapidly, with approximately 100,000 diagnoses and 7000 deaths per year in the US alone. We conducted a cross-sectional study with the aim of determining an association between the cost of care for invasive melanoma and the specialty involved in the treatment to adequately guide future treatment. Methods: We analyzed data from 3817 patients (2013–2018) using the Florida inpatient/outpatient dataset, CMS cost reports, and the National Plan and Provider Enumeration System. Covariates included age, sex, race/ethnicity, insurance type, region, county rurality, the number of procedures, the comorbidity index, obesity, metastatic cancer presence, hospital size, and physician volume. Multivariable mixed linear regression was used to analyze the data, and the cost was adjusted to the 2019 USD. Results: Dermatology had the largest decrease in the overall and outpatient costs compared to general surgery, followed by plastic surgery. The inpatient costs for dermatology and plastic surgery were lower than those for general surgery, but not significantly so. Conclusions: The costs associated with surgical procedures may vary depending on the specialty of the physician treating the patient. Dermatology was associated with lower treatment costs for invasive melanoma compared to other specialties, indicating that physician specialty influences the cost of care.

## 1. Introduction

Melanoma, which continues to be a fatal threat to patients in the United States and around the world, is a type of skin cancer that develops from the malignant transformation of pigment-producing melanocytes located in the basal layer of the epidermis [1,2]. Melanomas usually manifest on the skin; however, given that melanocytes are derived from neural crest cells, they can also develop in other regions such as the brain and gastrointestinal tract [3,4].

Melanoma is the third most common skin malignancy and ranks as the fifth and sixth most common malignancy in males and females, respectively. The frequency of primary cutaneous melanoma has been consistently increasing for several decades, and it continues to be the deadliest type of skin cancer [5,6]. In fact, the incidence of melanoma is growing so fast that it is second only to lung cancer in women. Every year in the Unites States, an estimated 100.000, diagnoses of melanoma are made, and approximately 7000 patients die of this disease each year [6].

The current literature has established melanoma to be a multifactorial disease, with a number of established risk factors that include environmental, genetic, and immunologic factors [7,8,9]. Melanoma has generally been considered among the most resistant cancers to conventional therapies, including chemotherapy, radiation therapy, and some experimental targeted diseases. However, a large improvement in patient life quality and average survivability has been achieved with incoming new immunotherapies specifically targeting patients with metastatic melanoma [10]. Nevertheless, researchers continue to struggle to uncover the base of the therapeutic resistance and relapse of melanoma [10].

The early treatment of melanoma is crucial due to the high fatality rate of metastasis. Treatment options include local wide excision, sentinel lymph node biopsy, elective node dissection, or combinations thereof [11]. Numerous research papers have investigated and documented the cost of care for patients with invasive melanoma, analyzing its correlation with patient demographics and other point-of-care characteristics. For instance, according to a study by the American Academy of Dermatology, the total medical cost for all types of melanoma in the U.S. was USD 1.4 billion in 2013 [12,13]. However, no study has investigated the correlation between the cost of care and the medical specialty responsible for treating melanoma. This study aims to investigate the association between the cost of care for invasive melanoma and the medical specialty responsible for its treatment by quantifying the differences in treatment costs across various specialties (including dermatology, surgical oncology, plastic surgery, ENT, general surgery, and medicine), analyzing how these cost differences vary between inpatient and outpatient settings, and examining the influence of patient demographics, comorbidities, and hospital characteristics on treatment costs. By addressing these objectives, we aim to provide valuable insights that can guide future melanoma care strategies and potentially reduce healthcare costs while maintaining high-quality patient outcomes. This knowledge is particularly important in providing patients with appropriate treatment guidance and impacting the economics of melanoma care.

## 2. Methods

### 2.1. Database and Study Population

We conducted an analysis using three data sources: the 2013–2018 Florida inpatient/outpatient dataset (FIOD) from the Agency for Health Care Administration (AHCA), the CMS cost reports, and the National Plan and Provider Enumeration System (NPPES). The FIOD provides data on all hospital discharges throughout Florida. The CMS cost reports offer information on hospital characteristics, utilization data, financial information, costs, and charges by cost center. The NPPES includes physicians’ National Provider Identifier (NPI) details, such as their provider name, specialty, and practice address.

Our study included patients from 2013 to 2018 who were diagnosed with invasive melanoma and underwent a related procedure. We excluded patients who were under 65 years old and on Medicare, those who were neither white, Black/African American, nor of Latino or Hispanic ethnicity, and those with a length of stay exceeding 14 days. After applying these exclusion criteria, our final study population comprised 3817 patients.

### 2.2. Dependent Variable

To explain the dependent variable—the log-transformed total cost of treatment for invasive melanoma—we employed a multivariate mixed linear regression model that estimated the unique contribution of each independent variable while accounting for variability across the hospitals. The log transformation of the cost was applied to address its skewed distribution, allowing us to interpret relationships in terms of percentage changes in costs.

Each independent variable in the model (e.g., physician specialty, patient demographics, and hospital characteristics) was selected based on its potential to impact treatment costs. For instance, physician specialty could influence costs due to differences in treatment protocols, resource utilization, and settings specific to specialties like dermatology, plastic surgery, and general surgery. Similarly, patient demographics (such as age and sex) and clinical factors (such as the comorbidity index and number of procedures) offer insights into how individual health characteristics and treatment complexity contribute to total costs.

By including these variables in the model, we were able to estimate their distinct effects on the dependent variable while controlling for other covariates. For example, this approach allows us to determine how much less or more costly treatment is when provided by a dermatologist rather than a general surgeon, independent of other factors like patient age or hospital size. The model also incorporates random intercepts for each hospital, capturing variations in costs due to hospital-specific practices or resources. This adjustment enhances the precision of our estimates, providing a clearer view of the unique impact of each variable on treatment costs.

### 2.3. Independent Variables

Patient-level covariates: age, sex, race/ethnicity, insurance type, region, county rurality, comorbidity index (Elixhauser score), obesity, and the presence of metastatic cancer.

Physician specialty: categorized as surgical oncology, plastic surgery, ENT, general surgery, dermatology, and medicine. General surgery was used as the reference category.

Hospital-level covariates: hospital size (small, medium, or large) and physician volume (measured as the number of melanoma-related surgical cases per physician).

Treatment characteristics: the number of procedures performed on each patient and the treatment setting (inpatient vs. outpatient).

We exponentiated parameter estimates and their 95% confidence intervals to interpret the percentage differences in the costs associated with each independent variable. The model included random intercepts for each hospital facility to account for clustering within hospitals, controlling for variations that may arise from hospital-specific practices.

### 2.4. Statistical Analysis

We performed descriptive statistics for the patient and physician characteristics. The continuous variables were summarized using the median and range, while the categorical variables were summarized using frequencies and percentages. Due to the non-normal distribution of costs, we log-transformed the cost data. Multivariable mixed linear regression was subsequently used to determine an association between the provider characteristics and the log average cost of a patient’s hospital treatment. Further, three models were run to identify differences: (1) overall (including both inpatient and outpatient costs), (2) inpatient costs alone, and (3) outpatient costs alone. We exponentiated parameter estimates and 95% confidence intervals to interpret percent differences.

We applied a multivariate mixed linear regression model to examine the association between the physician specialty and treatment costs for invasive melanoma, adjusting for relevant patient- and hospital-level covariates. The dependent variable was the log-transformed total cost, which includes both inpatient and outpatient expenses, adjusted to 2019 USD.

## 3. Results

Using the multivariate mixed linear regression model, we found that certain physician specialties were significantly associated with lower treatment costs for invasive melanoma. Compared to the reference category of general surgery, dermatology showed the largest decrease in total costs, with an estimated reduction of 88.49% (*p* < 0.0001). Plastic surgery was also associated with a significant cost reduction of 11.87% (*p* = 0.01). These findings were consistent in both inpatient and outpatient settings, where dermatology and plastic surgery incurred lower costs than general surgery.

Additional significant covariates included the patients’ sex, with the female patients incurring 8.32% lower costs than the males (*p* < 0.0001), and age, where the costs decreased by 2.15% for every 10-year increase in the patient age (*p* = 0.01). The patients from central Florida and southeast Florida experienced cost reductions of 11.03% (*p* = 0.04) and 10.81% (*p* = 0.05), respectively.

Treatment complexity, represented by the number of procedures a patient received, was strongly associated with higher costs, increasing the total costs by 20.31% per additional procedure (*p* < 0.0001). Similarly, inpatient services were associated with a substantial increase in total costs, estimated at 108.51% higher than outpatient services (*p* < 0.0001).

These results suggest that physician specialty and specific patient demographics have a statistically significant impact on treatment costs for invasive melanoma, underscoring the importance of specialty-specific practices and demographic factors in healthcare costs.

The total number of patients included in our study was 3483 (Table 1). The mean and median total cost of stay were USD 6723.3 (SD: USD 7632.1) and USD 5194.0.

Out of the 3483 patients, general surgery was the most common treating specialty (36.3%), followed by surgical oncology (27.2%) and plastic surgery (23.2%). The mean total cost was USD 6723.30 (SD: USD 7632.10). Our regression analysis showed that dermatology was associated with the largest decrease in overall and outpatient costs compared to general surgery, followed by plastic surgery. The majority of surgeries (33.4%) were performed in 2015, with 2014 (26.1%) and 2017 (16.5%) following closely behind. Females accounted for 38.6% of the patients, and males accounted for 61.4%. The mean age of the patients was 66.3 years (SD = 14.8).

Medicare was the most frequent patient payer, with a share of 35.1% of the patients, closely followed by commercial insurance (38.1%), Medicare managed care (19.6%), other payers (4.7%), and Medicaid (2.5%). Among the patient counties, 96.7% of the patients were from urban areas, while 3.3% were from rural areas. Among race/ethnicity, white patients accounted for 93.3% of the surgeries, while Hispanic or Latino and Black or African American patients accounted for 5.7% and 1.0%, respectively.

The majority of the surgeries were performed in central Florida (38.1%), followed by southeast Florida (27.7%), west central Florida (8.9%), southwest Florida (12.0%), northeast Florida (6.7%), northwest Florida (2.8%), and south Florida (6.8%). The mean Elixhauser score was 0.6 (SD = 1.1), including a range of 0.0 to 9.0. Most of the patients did not experience complications (97.5%), while only 2.5% did.

The mean physician volume was 35.0 (SD = 36.6), and the mean number of procedures per patient was 2.6 (SD = 1.5), with a range of 1.0 to 18.0. The majority of the surgeries were performed in large hospitals (88.5%), followed by medium-sized hospitals (9.3%) and small hospitals (2.2%). Most of the surgeries were performed in an outpatient setting (75.1%), while 24.9% were performed in an inpatient setting.

We explored the overall costs (Table 2), inpatient costs (Table 3), and outpatient costs (Table 4), while taking into account the percentage change in the total cost while adjusting for patient, facility, and physician characteristics.

### 3.1. Overall Costs

Dermatology exhibited the largest decrease in total costs compared to the reference group (general surgery), with an estimated reduction of 88.49% (*p* < 0.0001). Plastic surgery also demonstrated a significant decrease in total costs, with an estimate of 11.87% (*p* = 0.01).

The female patients incurred significantly lower total costs than the male patients, with an estimated decrease of 8.32% (*p* < 0.0001). Additionally, for every 10-year increase in age, the total costs decreased by 2.15% (*p* = 0.01). The patients in central Florida and southeast Florida experienced cost reductions of 11.03% and 10.81%, respectively.

The number of procedures a patient received was strongly positively associated with the total costs, increasing by 20.31% per additional procedure (*p* < 0.0001), highlighting the impact of care complexity on costs. The inpatient services were associated with a substantial increase in the total costs, estimated at 108.51% higher than the outpatient services (*p* < 0.0001).

### 3.2. Inpatient Costs

Several variables significantly affected the inpatient costs. The patients treated by dermatologists had costs 40.32% lower than those treated by general surgeons (*p* = 0.01). The plastic surgery patients also had lower costs, with a reduction of 15.39% (*p* = 0.04). The female patients incurred 11.16% lower costs than the male patients (*p* < 0.001). In contrast, the Hispanic or Latino patients experienced a 17.20% increase in costs compared to the white patients (*p* = 0.01). Each additional procedure increased the inpatient costs by 16.58% (*p* < 0.0001).

### 3.3. Outpatient Costs

Dermatology was associated with a 96.65% lower cost compared to general surgery (*p* < 0.0001). Medicine was significantly associated with a 49% cost reduction (*p* < 0.0001). Plastic surgery also demonstrated a cost reduction of 11.84% (*p* < 0.01). The female patients had a 7.52% decrease in costs compared to the male patients (*p* < 0.0001). The years 2016 and 2017 were associated with cost increases of 8.09% (*p* = 0.01) and 9.86% (*p* < 0.01), respectively. A higher Elixhauser score was associated with a 3.81% increase in the outpatient costs (*p* < 0.001). Additionally, each extra procedure received by the patient increased the outpatient costs by 25.09% (*p* < 0.0001). Lastly, for every 10-year increase in age, the costs decreased by 2.05% (*p* = 0.02).

## 4. Discussion

Skin cancer is the most common type of cancer in the United States [14,15]. While non-melanoma skin cancer is the most prevalent, with an incidence of 3.5 million patients in 2006 [14,16], melanoma skin cancer is the most deadly form, accounting for 59,695 new diagnoses and 8623 deaths in 2008 [17,18]. Alarmingly, the incidence of melanoma has been increasing at a rate of 3–7% per year [19,20]. 

Previous studies have assessed the economic burden of skin cancer using measures such as direct medical costs and indirect costs to analyze the cost-effectiveness of non-melanoma skin cancer (NMSC) treatments [12,21,22]. Higashi et al. reported annual Medicare payments of USD 562 million for NMSC and USD 202 million for actinic keratosis in 1998 USD. While these studies established important baseline cost data, they primarily focused on NMSC rather than melanoma, creating a knowledge gap in melanoma-specific cost analysis [21]. Our study addresses this gap by providing a more recent and specific analysis of invasive melanoma costs, demonstrating how treatment costs vary significantly by specialty, a factor not previously examined in depth. The systematic review by Guy Jr. et al. revealed that melanoma treatment costs are highly variable, ranging from USD 44.9 million among Medicare patients with existing cases to USD 932.5 million among newly diagnosed cases across all age groups. Their findings highlighted how costs vary by the phase of care and healthcare settings, but did not specifically examine the impact of physician specialty. Our results extend their work by demonstrating that specialty choice significantly influences these cost variations, with dermatology showing an 88.49% reduction in the overall costs compared to general surgery. Our findings particularly align with and build upon those of Burningham et al. [23], who examined melanoma in situ treatment cost across specialties at a single institution. They found that the mean total cost for melanoma in situ treated with wide local excision by dermatologists was USD 1089, compared to USD 5172 when treated by other specialties, a cost reduction of approximately 79%. This is comparable to our finding of an 88.49% reduction in the overall costs for invasive melanoma treated by dermatologists compared to general surgeons. While their study focused on melanoma in situ in a single setting, our research demonstrates similar cost advantages for dermatology across multiple healthcare settings and for invasive melanoma, suggesting that this cost benefit extends beyond early-stage disease. Furthermore, our study reveals that this cost advantage persists even after adjusting for multiple confounding factors not considered in previous studies, such as patient demographics, comorbidities, and hospital characteristics. 

Our analysis showed that patients treated by dermatology and plastic surgery had significantly lower overall costs than those treated by general surgery. This can be attributed to several factors reflecting fundamental differences in practice patterns and treatment approaches. Dermatologists often prioritize early detection and routine surveillance, leading to more cost-effective disease management. This aligns with Alexandrescu’s observation that early-stage treatment costs are primarily driven by initial tumor management and surveillance [24]. In contrast, surgical oncologists and general surgeons frequently manage more complex cases requiring extensive surgeries and prolonged hospital stays. Our data confirms this, highlighting a 108.51% higher cost associated with the inpatient services for these specialties The inpatient services were associated with a significant increase in the total cost, indicating that the treatment setting substantially impacts expenses. This finding suggests that specialty-specific protocols, particularly the decision to treat patients in inpatient versus outpatient settings, can significantly affect the total cost of care. Our inpatient cost analysis revealed similar trends, with dermatology and plastic surgery incurring lower costs than general surgery, and the female patients having lower costs than the males. The patients who underwent more procedures had higher inpatient costs, reflecting an increased case complexity. These findings emphasize the importance of considering healthcare settings and specialty-specific practices when evaluating cost-effectiveness. Furthermore, patients treated by surgical oncologists may incur a higher costs due to the utilization of expensive immunotherapies and targeted therapies, particularly for advanced melanoma [25], as well as comprehensive diagnostic testing.

In terms of the outpatient costs, the patients treated by dermatology, medicine, and plastic surgery had significantly lower expenses than those treated by general surgery. The female and older patients continued to incur lower costs, which is consistent with Behbani et al. [26], who suggest that earlier detection in females, potentially due to biological differences, may lead to more effective and less expensive treatments. The patients undergoing more procedures had higher outpatient costs due to increased costs for facility use, staff, supplies, and additional tests. Each additional procedure was associated with a 20.31% increase in total costs (*p* < 0.0001), a consistent trend across all the specialties. This highlights the complexity and intensity of care as key contributors to overall costs. Efforts to optimize the number of necessary procedures could enhance cost-effectiveness without compromising patient outcomes, thereby improving healthcare access.

Notably, the years 2016 and 2017 were associated with increased outpatient costs, possibly due to changes in healthcare policies or treatment practices during that period. These findings suggest that dermatological treatments for invasive melanoma may utilize less resource-intensive procedures in outpatient care, contributing to significant cost savings.

The Elixhauser score was associated with a 3.87% increase in the total cost per one-point increase (*p* < 0.0001), indicating that specific comorbidities, such as metastatic cancer and obesity, significantly elevate treatment costs across all the specialties. This underscores the need for tailored approaches to managing patients with complex medical histories. Even in cost-effective specialties like dermatology, certain comorbidities can negate these savings, suggesting that treatment strategies should be adapted to better address these patients’ needs.

Our findings suggest that healthcare systems may need to reevaluate their approach to melanoma care. While cost should not be the sole determining factor in treatment decisions, it is important in the context of value-based care. Based on our results, healthcare systems might consider prioritizing referrals to dermatologists or plastic surgeons for melanoma treatment when appropriate, given the potential for cost savings. However, this recommendation must be balanced with other factors such as physician expertise, case complexity, and patient outcomes. Future research should investigate the mechanisms behind the cost savings observed in dermatology, particularly in outpatient care, and whether these cost differences are associated with variations in treatment quality, patient satisfaction, or long-term outcomes. Understanding how dermatologists achieve these reductions could inform the best practices for cost management in other specialties.

Additionally, these findings could inform the development of new clinical guidelines for melanoma treatment. Professional organizations might consider recommending a multidisciplinary approach that leverages the cost-effectiveness of dermatology and plastic surgery while ensuring that patients have access to comprehensive expertise. Creating standardized protocols to determine which cases are best suited for each specialty could lead to more efficient resource allocation and improved patient care.

Furthermore, policymakers and insurance providers might use this information to design reimbursement structures that incentivize cost-effective care without compromising quality. This could include bundled payment models or ‘centers of excellence’ designations that encourage collaboration between specialties and reward efficient, high-quality care.

Implementing such changes would require careful consideration and further research. Factors such as regional variations in healthcare delivery, the availability of specialists, and individual patient needs must be taken into account. Future studies should aim to replicate these findings across different healthcare systems and geographic regions and explore the reasons behind the cost differences observed between specialties.

Overall, our findings indicate that different specialties result in varying costs for the treatment of invasive melanoma. Dermatology had the largest decrease in the overall, outpatient, and inpatient costs compared to general surgery, followed by plastic surgery. The patients treated by surgical oncology did not show a significant difference in costs compared to general surgery, despite representing a substantial proportion of the surgeries.

## 5. Limitations

Our study has several limitations. Its retrospective design and reliance on data from a single state may limit its generalizability. We excluded certain patient groups, such as those under 65 on Medicare, which might affect the applicability of our findings. We did not analyze the influence of different treatment modalities or stages of melanoma, nor did we compare various insurance plans, such as commercial and private insurance, which could have distinct cost structures. We also did not explore the role of institutional factors, such as hospital size and physician volume, in influencing costs. Investigating these factors may offer valuable insights into optimizing care delivery systems for greater efficiency. Future research should include a broader range of insurance types and age groups to provide a more comprehensive understanding of how insurance affects melanoma treatment costs.

## 6. Conclusions

Our study highlights the statistically significant impact of physician specialty on the treatment costs of invasive melanoma. Dermatology exhibited the most substantial cost reduction, with overall costs 88.49% lower than those associated with general surgery (*p* < 0.0001), followed by plastic surgery, with an 11.87% reduction (*p* = 0.01). These cost differences were evident in both the inpatient and outpatient settings, underscoring that the treating physician’s specialty considerably influences healthcare expenditures. 

Additionally, the patient demographics and care characteristics played a statistically significant role in the cost variations. The female patients incurred 8.32% lower costs than the males (*p* < 0.0001), and for every 10-year increase in age, the costs decreased by 2.15% (*p* = 0.01). Conversely, each additional procedure increased the overall costs by 20.31% (*p* < 0.0001), and the inpatient services were associated with a 108.51% increase in costs compared to the outpatient services (*p* < 0.0001).

These findings are significant as they offer essential guidance for optimizing patient treatment strategies and healthcare resource allocation. By identifying specialties associated with lower treatment costs, healthcare systems can consider strategic referrals to dermatology and plastic surgery when appropriate, potentially enhancing cost-effectiveness without compromising care quality. This study fills a gap in the literature by providing a detailed analysis of cost variations across specialties, contributing valuable insights to value-based care discussions.

However, our research focused solely on cost analysis and did not assess treatment outcomes or the quality of care. Future studies should explore the underlying reasons for these cost differences among specialties, including variations in treatment approaches, resource utilization, and efficiency. Investigating the relationship between cost and the quality of care is crucial to ensure that cost savings do not come at the expense of patient outcomes.

Understanding the interplay between physician specialty, treatment costs, and patient outcomes is increasingly important as healthcare systems strive for efficient and effective care delivery. Our findings suggest that leveraging cost-effective specialties could optimize care value, potentially reducing healthcare costs while maintaining or improving outcomes for patients with invasive melanoma. This research contributes to the broader effort of enhancing healthcare efficiency and effectiveness in oncology care.

## Figures and Tables

**Table 1:** Descriptive statistics.

	Total (*N* = 3483)
Total Cost of Stay	
N	3483
Mean (SD)	6723.3 (7632.1)
Median	5194.0
Q1, Q3	3525.6, 7438.0
Range	(27.5–195, 820.6)
Surgeon Group	
Surgical Oncology	946 (27.2%)
Plastic Surgery	809 (23.2%)
ENT	368 (10.6%)
General Surgery	1264 (36.3%)
Dermatology	38 (1.1%)
Medicine	58 (1.7%)
Year	
2013	206 (5.9%)
2014	908 (26.1%)
2015	1164 (33.4%)
2016	552 (15.8%)
2017	574 (16.5%)
2018	79 (2.3%)
Sex	
Female	1345 (38.6%)
Male	2138 (61.4%)
Age	
N	3483
Mean (SD)	66.3 (14.8)
Median	68.0
Q1, Q3	57.0, 77.0
Range	(12.0–100.0)
Patient Payer	
Medicare	1223 (35.1%)
Medicare Managed Care	684 (19.6%)
Medicaid	88 (2.5%)
Commercial	1326 (38.1%)
Other	162 (4.7%)
Patient County	
Missing	67
Rural	114 (3.3%)
Urban	3302 (96.7%)
Race	
Missing	129
White	3129 (93.3%)
Black or African American	35 (1.0%)
Hispanic or Latino	190 (5.7%)
Patient Region	
Southwest Florida	410 (12.0%)
Northeast Florida	229 (6.7%)
Northwest Florida	96 (2.8%)
Southeast Florida	843 (27.7%)
Central Florida	1303 (38.1%)
South Florida	232 (6.8%)
West Central Florida	303 (8.9%)
Elixhauser Score	
N	3483
Mean (SD)	0.6 (1.1)
Median	0.0
Q1, Q3	0.0, 1.0
Range	(0.0–9.0)
Complication	
No	3397 (97.5%)
Yes	86 (2.5%)
Physician Volume	
N	3483
Mean (SD)	35.0 (36.6)
Median	23.0
Q1, Q3	5.0, 52.0
Range	(1.0–157.0)
Number of Procedures	
N	3483
Mean (SD)	2.6 (1.5)
Median	2.0
Q1, Q3	2.0, 3.0
Range	(1.0–18.0)
Hospital Size	
Small	75 (2.2%)
Medium	324 (9.3%)
Large	3084 (88.5%)
Setting	
Inpatient	867 (24.9%)
Outpatient	2616 (75.1%)

**Table 2:** Mixed effect model—overall costs.

Parameter	Estimate	Lower 95% CI	Upper 95% CI	*p* Value
Surgical Group (General is Referent)				
Dermatology	−88.49	−91.33	−84.73	<0.0001
ENT	−1.16	−11.76	10.73	0.84
Medicine	−11.24	−24.08	3.78	0.14
Surgical Oncology	−7.78	−20.25	6.64	0.27
Plastic	−11.87	−19.84	−3.11	0.01
Female (Male is Referent)	−8.32	−11.09	−5.46	<0.0001
Year (2013 is Referent)				
2014	−0.97	−8.33	6.97	0.80
2015	−0.94	−8.30	7.00	0.81
2016	2.17	−6.07	11.13	0.62
2017	4.61	−3.95	13.94	0.30
2018	7.44	−5.49	22.14	0.27
Rural Patient (Urban is Referent)	−3.49	−12.76	6.77	0.49
Payer				
Medicaid	4.99	−5.12	16.17	0.35
Medicare	0.01	−4.83	5.09	1.00
Medicare Managed Care	0.37	−4.84	5.86	0.89
Other	0.71	−6.68	8.68	0.86
Elixhauser Score	3.87	2.12	5.65	<0.0001
Race/Ethnicity (White is Referent)				
Black or African American	−10.15	−23.06	4.94	0.18
Hispanic or Latino	5.57	−2.13	13.88	0.16
Patient Region (Northeast is Referent)				
Central Florida	−11.03	−20.28	−0.72	0.04
Northwest Florida	−6.16	−20.48	10.74	0.45
South Florida	−2.70	−15.26	11.53	0.69
Southeast Florida	−10.81	−20.59	0.18	0.05
Southwest Florida	−9.44	−19.28	1.59	0.09
West Central Florida	−10.29	−20.39	1.08	0.07
Age (10-unit Increase)	−2.15	−3.68	−0.60	0.01
Hospital Size (Small is Referent)				
Large	2.63	−14.73	23.52	0.78
Medium	−5.40	−22.38	15.28	0.58
Physician Surgical Count (10-unit Increase)	1.28	−1.17	3.78	0.31
Number of Procedures Patient Received	20.31	18.93	21.71	<0.0001
Setting (Outpatient is Referent)				
Inpatient	108.51	97.27	120.36	<0.0001

Percentage change in the total cost, adjusting for the patient, facility, and physician characteristics.

**Table 3:** Mixed effect model—inpatient costs.

Parameter	Estimate	Lower 95% CI	Upper 95% CI	*p* Value
Surgical Group (General is Referent)				
Dermatology	−40.32	−60.42	−10.02	0.01
ENT	−2.66	−16.60	13.61	0.73
Medicine	7.13	−11.04	29.01	0.47
Surgical Oncology	−7.07	−22.73	11.76	0.44
Plastic	−15.39	−27.69	−1.00	0.04
Female (Male is Referent)	−11.16	−17.01	−4.90	<0.001
Year (2013 is Referent)				
2014	0.60	−8.73	10.88	0.90
2015	3.08	−6.53	13.69	0.54
2016	−2.21	−12.94	9.85	0.71
2017	−3.01	−14.57	10.11	0.64
2018	4.12	−9.58	19.90	0.57
Rural Patient (Urban is Referent)	−11.18	−24.93	5.09	0.17
Payer				
Medicaid	8.03	−6.72	25.12	0.30
Medicare	−3.37	−13.17	7.53	0.53
Medicare Managed Care	−7.98	−17.78	3.00	0.15
Other	0.24	−11.64	13.71	0.97
Elixhauser Score	5.44	2.55	8.41	<0.001
Race/Ethnicity (White is Referent)				
Black or African American	2.53	−15.40	24.25	0.80
Hispanic or Latino	17.20	3.36	32.88	0.01
Patient Region (Northeast is Referent)	0.00	0.00	0.00	
Central Florida	−8.67	−21.68	6.50	0.25
Northwest Florida	−6.65	−24.29	15.10	0.52
South Florida	−4.14	−20.78	15.99	0.66
Southeast Florida	−3.41	−17.61	13.25	0.67
Southwest Florida	−7.37	−20.97	8.57	0.34
West Central Florida	−8.51	−21.95	7.24	0.27
Age (10-unit Increase)	−3.08	−6.17	0.12	0.06
Hospital Size (Small is Referent)				
Large	0.54	−32.67	50.11	0.98
Medium	14.92	−26.10	78.75	0.54
Physician Surgical Count (10-unit Increase)	1.11	−1.91	4.23	0.47
Number of Procedures Patient Received	16.58	14.65	18.54	<0.0001

Percentage change in the total cost adjusting for the patient, facility, and physician characteristics.

**Table 4:** Mixed effect model—outpatient costs.

Parameter	Estimate	Lower 95% CI	Upper 95% CI	*p* Value
Surgical Group (General is Referent)				
Dermatology	−96.65	−97.58	−95.36	<0.0001
ENT	2.73	−9.54	16.66	0.68
Medicine	−49.00	−61.69	−32.10	<0.0001
Surgical Oncology	−10.10	−23.10	5.11	0.18
Plastic	−11.84	−19.68	−3.24	<0.01
Female (Male is Referent)	−7.52	−10.54	−4.41	<0.0001
Year (2014 is Referent)				
2015	1.45	−2.67	5.74	0.50
2016	8.09	1.83	14.73	0.01
2017	9.86	3.49	16.63	<0.01
Rural Patient (Urban is Referent)	1.28	−10.17	14.19	0.84
Payer				
Medicaid	−11.70	−23.32	1.69	0.08
Medicare	1.14	−4.21	6.78	0.68
Medicare Managed Care	5.55	−0.43	11.91	0.07
Other	6.66	−2.85	17.09	0.18
Elixhauser Score	3.81	1.58	6.08	<0.001
Race/Ethnicity (White is Referent)				
Black or African American	−25.20	−46.84	5.25	0.10
Hispanic or Latino	−0.31	−9.05	9.26	0.95
Patient Region (Northeast is Referent)				
Central Florida	−7.57	−19.06	5.55	0.24
Northwest Florida	11.33	−12.04	40.89	0.37
South Florida	8.59	−8.62	29.03	0.35
Southeast Florida	−10.52	−22.24	2.98	0.12
Southwest Florida	−4.93	−17.45	9.49	0.48
West Central Florida	−9.41	−22.50	5.88	0.21
Age (10-unit Increase)	−2.05	−3.74	−0.34	0.02
Hospital Size (Small is Referent)				
Large	4.27	−13.52	25.70	0.66
Medium	−9.55	−25.84	10.30	0.32
Physician Surgical Count (10-unit Increase)	1.62	−0.63	3.92	0.16
Number of Procedures Patient Received	25.09	23.11	27.11	<0.0001

Percentage change in the total cost adjusting for the patient, facility and physician characteristics.

## Data Availability

The original data presented in the study are openly available from the Florida Agency for Health Care Administration’s secure data portal at https://quality.healthfinder.fl.gov/ (accessed on 6 August 2024) and from the Centers for Medicare & Medicaid Services at https://nppes.cms.hhs.gov/#/ (accessed on 6 August 2024) and https://www.cms.gov/Research-Statistics-Data-and-Systems/Downloadable-Public-Use-Files/Cost-Reports (accessed on 6 August 2024).
